# A novel phylogeny and morphological reconstruction of the *PIN* genes and first phylogeny of the ACC-oxidases (*ACOs*)

**DOI:** 10.3389/fpls.2014.00296

**Published:** 2014-06-24

**Authors:** Ronald M. Clouse, Nicola Carraro

**Affiliations:** ^1^Department of Bioinformatics and Genomics, University of North Carolina at CharlotteCharlotte, NC, USA; ^2^Department of Agronomy, Purdue UniversityWest Lafayette, IN, USA

**Keywords:** ethylene synthesis, auxin, ancestral reconstruction, GC mutational bias, PIN-formed, 1-aminocyclopropane-1-carboxylate oxidase

## Abstract

The *PIN* and *ACO* gene families present interesting questions about the evolution of plant physiology, including testing hypotheses about the ecological drivers of their diversification and whether unrelated genes have been recruited for similar functions. The PIN-formed proteins contribute to the polar transport of auxin, a hormone which regulates plant growth and development. *PIN* loci are categorized into groups according to their protein length and structure, as well as subcellular localization. An interesting question with *PIN* genes is the nature of the ancestral form and location. *ACO*s are members of a superfamily of oxygenases and oxidases that catalyze the last step of ethylene synthesis, which regulates many aspects of the plant life cycle. We used publicly available *PIN* and *ACO* sequences to conduct phylogenetic analyses. Third codon positions of these genes in monocots have a high GC content, which could be historical but is more likely due to a mutational bias. Thus, we developed methods to extract phylogenetic information from nucleotide sequences while avoiding this convergent feature. One method consisted in using only A-T transformations, and another used only the first and second codon positions for serine, which can only take A or T and G or C, respectively. We also conducted tree-searches for both gene families using unaligned amino acid sequences and dynamic homology. *PIN* genes appear to have diversified earlier than *ACOs*, with monocot and dicot copies more mixed in the phylogeny. However, gymnosperm *PINs* appear to be derived and not closely related to those from primitive plants. We find strong support for a long *PIN* gene ancestor with short forms subsequently evolving one or more times. *ACO* genes appear to have diversified mostly since the dicot-monocot split, as most genes cluster into a small number of monocot and dicot clades when the tree is rooted by genes from mosses. Gymnosperm *ACO*s were recovered as closely related and derived.

## Introduction

The dramatic increase in the amount of publicly available genomic information has facilitated analyses of gene-family origins and evolution. Plant gene phylogenies have proliferated, but they are commonly made using distance matrices of amino acid sequences. This method is expected to amplify misleading information resulting from convergence (Farris, 1983; Simmons, 2000; Simmons et al., 2002), obscuring any signal of the true history.

Methodological approaches to studying gene family histories need focused attention, since gene phylogenies are inherently difficult to verify. Molecular phylogenies of species can be compared to their morphology and biogeography to ask whether resulting trees are plausible. Perfect congruence is not expected, but phylogenies at extreme variance cast doubt on the quality of the methods and underlying data. Because gene copies can undergo any combination of diversification, loss, and neofunctionalization in different taxonomic lineages, nearly any sort of phylogenetic hypothesis can be the reasonable outcome of an analysis. Gene copies in the same taxon or with the same morphology would be expected to have a higher probability of being closely related, but this is not necessarily so, and it is dependent on the timing of gene family diversification events and the phylogenetic placement of progenitor copies.

Here we employ phylogenetic methods designed to minimize the effects of convergence and amplify historical signal in the date. We apply these methods to the *PIN*-formed (*PIN*) auxin transporters and the ethylene-forming, ACC-oxidase enzymes (*ACO*). We chose to analyze these gene families because both are important plant development genes for which there are hypotheses about the timing and drivers of their diversification, but they differ in the amount of phylogenetic study thus far received. The *PIN* genes have been the subject of different phylogenetic analyses (Paponov et al., 2005; Křeček et al., 2009; De Smet et al., 2011; Carraro et al., 2012; Viaene et al., 2013; Bennett et al., 2014), but for *ACO*s no such analysis has been attempted. We thus are able to compare the results of our methods to prior analyses in *PIN* genes, and we lay the groundwork for a new discussion on the history of *ACO*s.

PIN-formed proteins polarly transport the plant hormone auxin, which regulates several aspects of plant growth and development (Robert and Friml, 2009; Zazímalová et al., 2010). Since the discovery and characterization of the first *PIN* mutant in *Arabidopsis* (Okada et al., 1991), several other *PINs* have been identified and characterized in different plant species. In the *Arabidopsis* genome, there are eight *PIN* loci, which are categorized into groups according to their protein length and structure, as well as subcellular localization (Paponov et al., 2005; Křeček et al., 2009). The first cladistic analysis of *PIN* genes (Carraro et al., 2012), which was rooted by moss and lycophyte copies, suggested that *PIN* genes diversified mostly since the rise of land plants, around the time of the monocot-dictot split. A subsequent analysis (Viaene et al., 2013) focused on the evolution of *PIN* gene morphology; the preferred topology—rooted by protist, animal, bacterial genes—suggested that the moss sequence “PpPIN1D” is sister to all other *PIN* genes, and the morphology evolved from short forms to long.

*ACO*s help in the synthesis of ethylene, which is a gaseous hormone under normal environmental conditions, and which regulates many aspects of the plant life cycle (Bleecker and Kende, 2000; Lin et al., 2009). In higher plants, ethylene is synthesized via two committed enzyme-catalyzed steps from S-adenosyl-L-methionine. The first step is catalyzed by 1-aminocyclopropane-1-carboxylic acid (*ACC*) synthase (ACS), and the second (and last) step is carried out by *ACC* oxidase (*ACO*). *ACO*s are members of a superfamily of oxygenases and oxidases, most of which utilize Fe (II) as a cofactor and 2-oxoglutarate (*2OG*) as a cosubstrate (Sato and Theologis, 1989; Bidonde et al., 1998; Wang et al., 2002). The subcellular localization of *ACO* proteins is prevalently cytosolic rather than membrane-bound (Chung et al., 2002; Hudgins et al., 2006; Lin et al., 2009). *ACO*s have high similarity throughout the protein coding sequences and expression analyses reveal that the *ACO* genes display a high degree of differential expression in tissues at various stages of development. A variety of plant species produce ethylene, including unicellular and multicellular algae, although angiosperms use a different biosynthetic pathway from primitive land plants and algae (Wang et al., 2002; De Paepe and Van der Straeten, 2005; Plettner et al., 2005; Yordanova et al., 2010; Wanke, 2011; Yasumura et al., 2012).

## Materials and methods

### Assembling of the *ACO* and *PIN* data sets

For all phylogenetic analyses, unless otherwise specified, coding sequences (CDS) were used. All taxa with publicly available sequences were included, although a random subset of all available angiosperm sequences were taken, so as to generate a manageable data set size. For the *PIN* data set (Table 1), reported unique sequence identifiers were used to retrieve the corresponding sequences from the Phytozome v.9.1 (www.phytozome.org) (Goodstein et al., 2012), ConGenIE (congenie.org), and Genebank (www.ncbi.nlm.nih.gov/genbank/) (Benson et al., 2005) databases. The only major plant group that was not included in the *PIN* data set was ferns, as previously reported *PIN* genes from them are not publicly available (Viaene et al., 2013). *ACO* sequences (Table 2) were identified from previously published studies and via queries with the BLASTn algorithm at the National Centre for Biotechnology Information (NCBI) nucleotide collection and from the Phytozome v.9.1 (Goodstein et al., 2012). Only proteins that were annotated as aminocyclopropane-carboxylate oxidases were retained. Transmembrane profiles for *PIN* amino acid sequences were predicted querying the TMHMM Server v.2.0 (www.cbs.dtu.dk/services/TMHMM/) (Moller et al., 2001). *PIN* proteins were classified (1–5) according to their length, number of transmembrane domains, and length of the central hydrophilic loop (See Table 1). Generally, caution should be exerted when classifying *PIN* proteins according to their number of TMDs, as those are predicted protein domains that will need final confirmation by reconstruction of the tertiary structure by crystallography. In two cases where gene sequences showed no notable differences from long forms but were predicted to have only the N-terminal trans-membrane domain (OsPIN3a and Aco018694), we coded them as long.

**Table 1:** ***PIN* genes analyzed in this study**.

**Species**	**Gene code**	**Terminal name**	**Protein (aa)**	**TMDs**	**Type**	**Score**
*Aquilegia caerulea*	AcoGoldSmith_v1.001931m.g	Aco001931	654	9	Long	1
	AcoGoldSmith_v1.007499m.g	Aco007499	356	9	Short	2
	AcoGoldSmith_v1.016169m.g	Aco016169	620	9	Long	1
	AcoGoldSmith_v1.018139m.g	Aco018139	641	9	Long	1
	AcoGoldSmith_v1.018694m.g	Aco018694	612	5	Long	1
*Arabidopsis thaliana*	AT1G73590	AtPIN1	622	9	Long	1
	AT5G57090	AtPIN2	647	9	Long	1
	AT1G70940	AtPIN3	640	9	Long	1
	AT2G01420	AtPIN4	616	10	Long	1
	AT5G16530	AtPIN5	351	9	Short	2
	AT1G77110	AtPIN6	570	9	Reduced	3
	AT1G23080	AtPIN7	619	9	Long	1
	AT5G15100	AtPIN8	367	8	Short	2
*Citrus sinensis*	Orange1.1g006199m.g	Csi_g006199	657	10	Long	1
	Orange1.1g007420m.g	Csi_g007420	604	8	Long	1
	Orange1.1g007826m.g	Csi_g007826	588	8	Long	1
	Orange1.1g018360m.g	Csi_g018360	357	8	Short	2
	Orange1.1g019021m.g	Csi_g019021	347	9	Short	2
	Orange1.1g035534m.g	Csi_g035534	291	5	N-terminal TMD only	5
	Orange1.1g036474m.g	Csi_g036474	646	9	Long	1
	Orange1.1g041301m.g	Csi_g041301	291	7	Short	2
	Orange1.1g048649m.g	Csi_g048649	256	5	N-terminal TMD only	5
*Eucalyptus grandis*	Eucgr.A02229	EgrA02229_1	599	8	Long	1
	Eucgr.B00948	EgrB00948_1	587	9	Long	1
	Eucgr.B01403	EgrB01403_1	365	9	Short	2
	Eucgr.B01405	EgrB01405_1	364	9	Short	2
	Eucgr.B01406	EgrB01406_1	285	7	Short	2
	Eucgr.B02902	EgrB02902_1	657	9	Long	1
	Eucgr.C00078	EgrC00078_1	626	9	Long	1
	Eucgr.F04265	EgrF04265_1	530	8	Reduced	3
	Eucgr.G02187	EgrG02187_1	652	9	Long	1
	Eucgr.G02548	EgrG02548_1	338	9	Short	2
	Eucgr.G02549	EgrG02549_1	360	9	Short	2
	Eucgr.H01382	EgrH01382_1	262	6	Short	2
	Eucgr.H01390	EgrH01390_1	519	7	Long	1
	Eucgr.01919	EgrI01919_1	356	8	Short	2
	Eucgr.K02271	EgrK02271_1	598	9	Long	1
*Manihot esculenta*	Cassava4.1_003367m.g	Mes003367	646	9	Long	1
	Cassava4.1_003794m.g	Mes003794	614	8	Long	1
	Cassava4.1_003807m.g	Mes003807	614	8	Long	1
	Cassava4.1_006998m.g	Mes006998	468	9	Long	1
	Cassava4.1_010607m.g	Mes010607	357	9	Short	2
	Cassava4.1_010688m.g	Mes010688	354	9	Short	2
	Cassava4.1_026579m.g	Mes026579	598	7	Long	1
	Cassava4.1_029063m.g	Mes029063	361	8	Short	2
	Cassava4.1_029078m.g	Mes029078	626	9	Long	1
	Cassava4.1_030090m.g	Mes030090	380	5	N-terminal TMD only	5
	Cassava4.1_033391m.g	Mes033391	355	8	Short	2
*Medicago truncatula*	Medtr2g043210	Mtr2g043210	315	4	N-terminal TMD only	5
	Medtr4g154810	Mtr4g154810	524	8	Long	1
	Medtr6g083450	Mtr6g083450	659	10	Long	1
	Medtr7g008720	Mtr7g008720	357	8	Short	2
	Medtr7g089430	Mtr7g089430	363	9	Short	2
	Medtr7g106430	Mtr7g106430	591	8	Long	1
	Medtr8g130020	Mtr8g130020	625	9	Long	1
	Medtr8g130040	Mtr8g130040	568	10	Long	1
	Medtr4g084870	Mtr4g084870	659	10	Long	1
	MtrAAT48627	MtrAAT48627	527	9	Long	1
	MtrAY115838	MtrAY115838	621	10	Long	1
*Oryza sativa*	LOC_Os06g12610	OsPIN1a	595	10	Long	1
	LOC_Os02g50960	OsPIN1b	554	9	Reduced	3
	LOC_Os11g04190	OsPIN1c	592	10	Long	1
	LOC_Os12g04000	OsPIN1d	390	4	C-terminal TMD only	4
	LOC_Os06g44970	OsPIN2	630	9	Long	1
	LOC_Os01g45550	OsPIN3a	670	5	Long	1
	LOC_Os05g50140	OsPIN3b	591	10	Long	1
	LOC_Os01g69070	OsPIN5a	363	7	Short	2
	LOC_Os08g41720	OsPIN5b	398	7	Short	2
	LOC_Os09g32770	OsPIN5c	357	7	Short	2
	LOC_Os01g51780	OsPIN8	311	5	Short	2
	LOC_Os01g58860	OsPIN9	426	10	Reuced	3
*Physcomitrella patens*	Pp1s10_17V6.1	PpPIN1A	713	9	Long	1
	Pp1s18_186V6.1	PpPIN1B	713	9	Long	1
	Pp1s32_43V6.1	PpPIN1C	698	9	Long	1
*Picea abies*	FJ031883.2	PaPIN1	699	10	Long	1
	MA_61553g0010	PaPIN2	426	3	C-terminal TMD only	4
	MA_69724g0010	PaPIN3	625	8	Long	1
*Populus tomentosa*	AAP59843.1	PtoPIN1	619	7	Long	1
*Populus tremula × tremuloides*	AF190881.1	PttPIN1	614	9	Long	1
	AF515435.1	PttPIN2	640	9	Long	1
	AF515434.1	PttPIN3	588	9	Long	1
*Populus trichocarpa*	POPTR_0015s04570	PtrPIN1	614	9	Long	1
	POPTR_0016s03450	PtrPIN2	588	8	Long	1
	POPTR_0010s12320	PtrPIN3	645	9	Long	1
	POPTR_0005s20990	PtrPIN4	534	9	Long	1
	POPTR_0002s07310	PtrPIN5	532	8	Long	1
	POPTR_0008s12830	PtrPIN6	649	9	Long	1
	POPTR_0012s04470	PtrPIN7	609	9	Long	1
	POPTR_0006s03540	PtrPIN8	587	9	Long	1
	POPTR_0018s13610	PtrPIN9	633	9	Long	1
	POPTR_0001s21230	PtrPIN10	547	10	Long	1
	POPTR_0013s08510	PtrPIN11	346	9	Short	2
	POPTR_0019s07990	PtrPIN12	346	10	Short	2
	POPTR_0004s12310	PtrPIN13	355	8	Short	2
	POPTR_0017s11440	PtrPIN14	358	8	Short	2
	POPTR_0014s14390^a^	PtrPIN15	370	8	Short	2
	POPTR_0014s14390^a^	PtrPIN16	304	6	Short	2
*Prunus persicum*	ppa002528m.g	Ppe002528	662	10	Long	1
	ppa002944m.g	Ppe002944	619	9	Long	1
	ppa003159m.g	Ppe003159	597	8	Long	1
	ppa007621m.g	Ppe007621	361	9	Short	2
	ppa021573m.g	Ppe021573	357	7	Short	2
	ppa022797m.g	Ppe022797	550	9	Long	1
	ppa024134m.g	Ppe024134	649	9	Long	1
	ppa025174m.g	Ppe025174	602	8	Long	1
*Ricinus communis*	Rco27985.t000045	Rc27985	544	8	Long	1
	Rco29662.t000026	Rc29662	635	8	Long	1
	Rco29816.t000014	Rc29816	646	10	Long	1
	Rco29822.t000149	Rc29822	313	7	Short	2
	Rco30128.t000486	Rc30128	357	9	Short	2
	Rco30180.t000054	Rc30180	613	8	Long	1
*Selaginella moellendorrfii*	234325	SmPIN1-1	625	9	Long	1
	XM_002990455.1	SmPIN1-2	617	9	Long	1
	102666	SmPIN2-1	602	9	Long	1
	XM_002977411.1	SmPIN2-2	716	9	Long	1
	99301	SmPIN3-1	669	9	Long	1
	XM_002976656.1	SmPIN3-2	672	9	Long	1
	119024	SmPIN4-1	687	9	Long	1
	231064	SmPIN5-1	636	9	Long	1
	268490	SmPIN5-2	625	9	Long	1
*Sorghum bicolor*	Sb02g029210	SbPIN1	371	9	Short	2
	Sb03g029320	SbPIN2	653	8	Long	1
	Sb03g032850	SbPIN3	362	7	Short	2
	Sb03g037350	SbPIN4	444	10	Reduced	3
	Sb03g043960	SbPIN5	336	7	Short	2
	Sb04g028170	SbPIN6	605	10	Long	1
	Sb05g002150	SbPIN7	583	9	Long	1
	Sb07g026370	SbPIN8	402	9	Short	2
	Sb10g004430	SbPIN9	600	10	Long	1
	Sb10g008290	SbPIN10	606	9	Long	1
	Sb10g026300	SbPIN11	626	9	Long	1
*Vitis vinifera*	GSVIVG01025748001	VvPIN1a	604	8	Long	1
	GSVIVG01025749001	VvPIN1b	591	8	Long	1
	GSVIVG01029266001	VvPIN2	630	10	Long	1
	GSVIVG01019110001	VvPIN5b	361	9	Short	2
	GSVIVG01019126001	VvPIN5a	356	9	Short	2
	GSVIVG01010025001	VvPIN6	532	9	Reduced	3
	GSVIVG01033005001	VvPIN8	357	8	Short	2
	GSVIVG01031663001	VvPIN9	463	8	Reuced	3
*Zea mays*	GRMZM2G098643	ZmPIN1a	601	9	Long	1
	GRMZM2G074267	ZmPIN1b	595	8	Long	1
	GRMZM2G149184	ZmPIN1c	597	8	Long	1
	GRMZM2G171702_T01	ZmPIN1d	580	8	Long	1
	JQ421085.1	ZmPIN2	626	9	Long	1
	GRMZM2G025742	ZmPIN5a	382	9	Short	2
	GRMZM2G148648	ZmPIN5b	385	7	Short	2
	GRMZM2G040911	ZmPIN5c	365	7	Short	2
	GRMZM5G839411	ZmPIN8	359	7	Short	2
	GRMZM5G859099	ZmPIN9	433	10	Short	2
	GRMZM2G126260	ZmPIN10a	610	8	Long	1
	GRMZM2G160496	ZmPIN10b	581	8	Long	1

a These genes are distinct in GenBank but they retrieve the same entry in the phytozome database (www.phytozome.org).

**Table 2:** ***ACO* genes analyzed in this study**.

**Species**	**Gene code**	**Terminal name**
*Arabidopsis thaliana*	AT1G62380	AtACO2
	AT1G12010	AtACO3
	AT1G05010	AtACO4
	AT1G77330	AtACO5
	AT1G03400	AtACO6
	AT2G25450	AtACO7
	AT2G30830	AtACO8
	AT2G30840	AtACO9
	AT3G47190	AtACO10
	AT3G61400	AtACO11
	AT5G43440	AtACO12
	AT5G43450	AtACO13
*Carica papaya*	evm.model.supercontig_132.27	CpACO1
	evm.model.supercontig_64.148	CpACO2
*Eucalyptus grandis*	Eucgr.K00740	EgK00740
	Eucgr.K00750	EgK00750
	Eucgr.K00746	EgK00746
	Eucgr.K00749	EgK00749
	Eucgr.K00747	EgK00747
	Eucgr.C00906	EgC00906
	Eucgr.C03886	EgC03886
	Eucgr.F03839	EgF03839
*Glycine max*	Glyma07g39420	Gm07g39420
	Glyma17g01330	Gm17g01330
	Glyma09g01110	Gm09g01110
	Glyma15g11930	Gm15g11930
	Glyma14g05390	Gm14g05390
	Glyma02g43560	Gm02g43560
	Glyma06g12340	Gm06g12340
	Glyma04g42460	Gm04g42460
	Glyma05g36310	Gm05g36310
	Glyma07g15480	Gm07g15480
	Glyma08g03310	Gm08g03310
*Gossipium raimondii*	Gorai.010G184900	GrACO1
	Gorai.009G182300	GrACO2
	Gorai.004G062100	GrACO3
	Gorai.007G170100	GrACO4
	Gorai.001G096300	GrACO5
	Gorai.001G096400	GrACO6
	Gorai.001G011100	GrACO7
	Gorai.013G107500	GrACO8
*Malus domestica*	MDP0000195885	MdACO1
	MDP0000200737	MdACO2
	MDP0000725984	MdACO3
	MDP0000251295	MdACO4
	MDP0000453114	MdACO5
	MDP0000025650	MdACO6
	MDP0000200896	MdACO7
*Oryza sativa*	LOC_Os02g53180	Os02g53180
	LOC_Os09g27750	Os09g27750
	LOC_Os05g05680	Os05g05680
	LOC_Os05g05670	Os05g05670
	LOC_Os01g39860	Os01g39860
	LOC_Os11g08380	Os11g08380
	LOC_Os06g37590	Os06g37590
	LOC_Os09g27820	Os09g27820
*Physcomitrella patens*	Pp1s191_95V6	PpACO1
	Pp1s50_26V6	PpACO2
	Pp1s50_26V6	PpACO3
	Pp1s180_67V6	PpACO4
	Pp1s327_42V6	PpACO5
*Picea abies*	MA_2297g0010	PaACO1
	MA_9554510g0010	PaACO2
	MA_10431299g0010	PaACO3
	MA_10437223g0010	PaACO4
	MA_54476g0010	PaACO5
*Picea glauca*	DQ480741	PgACO1
*Picea sitchensis*	DQ480740	PsiACO1
	ABR17770	PsiACO2
*Pinus pinaster*	CBL95267	PpiACO1
*Pinus taeda*	GQ258776	PtdaACO1
	GQ258775	PtdaACO2
	GQ258774	PtdaACO3
*Pisum sativum*	AB128037	PsACO1
*Populus trichocarpa*	Potri002G224100	PtACO1
	Potri004G003000	PtACO2
	Potri011G020900	PtACO3
	Potri014G159000	PtACO4
	Potri.002G078600	PtACO5
	Potri.005G182700	PtACO6
	Potri.006G151600	PtACO7
*Pseudotsuga menziesii*	ABF20554	PsmACO1
*Selaginella moellendorffii*	116993	SmACO1
	407386	SmACO2
	169250	SmACO3
	228878	SmACO4
	117056	SmACO5
	402706	SmACO6
*Solanum lycopersicum*	Solyc06g060070	SlACO1
	Solyc12g005940	SlACO2
	Solyc07g049550	SlACO3
	Solyc07g026650	SlACO4
	Solyc07g049530	SlACO5
	Solyc02g081190	SlACO6
	Solyc02g036350	SlACO7
*Sorghum bicolor*	Sb02g026280	Sb02g026280
	Sb05g005710	Sb05g005710
	Sb05g005720	Sb05g005720
	Sb09g003790	Sb09g003790
	Sb09g003800	Sb09g003800
	Sb10g022640	Sb10g022640
	Sb04g034520	Sb04g034520
*Zea mays*	GRMZM2G052422	Zm2G052422
	GRMZM2G072529	Zm2G072529
	GRMZM2G126732_T02	Zm2G126732
	GRMZM2G164883	Zm2G164883
	GRMZM2G166616	Zm2G166616
	GRMZM2G166639_T01	Zm2G166639_T01
	GRMZM2G166639_T02	Zm2G166639_T02
	GRMZM2G332423	Zm2G332423

### Alignment and phylogenetic analysis

The moss *PIN* gene “PpPIN1D” (Gene Code “Pp1s79_126V6.1”) was excluded from analysis due to suspicion of it being a pseudogene; although not the most distant gene in the data set, it has only about half the number of nucleotides relative to other moss *PIN* genes (with the gaps appearing in the middle), and in preliminary phylogenies it was recovered on a very long branch (usually twice as long as its sister). This can also be seen in our previous phylogeny of *PIN* genes (Figure 2A in Carraro et al., 2012). Likewise, we did not include purported *PIN* homologs from non-plants (Viaene et al., 2013), as we had no evidence for their homology.

**Figure 1:**
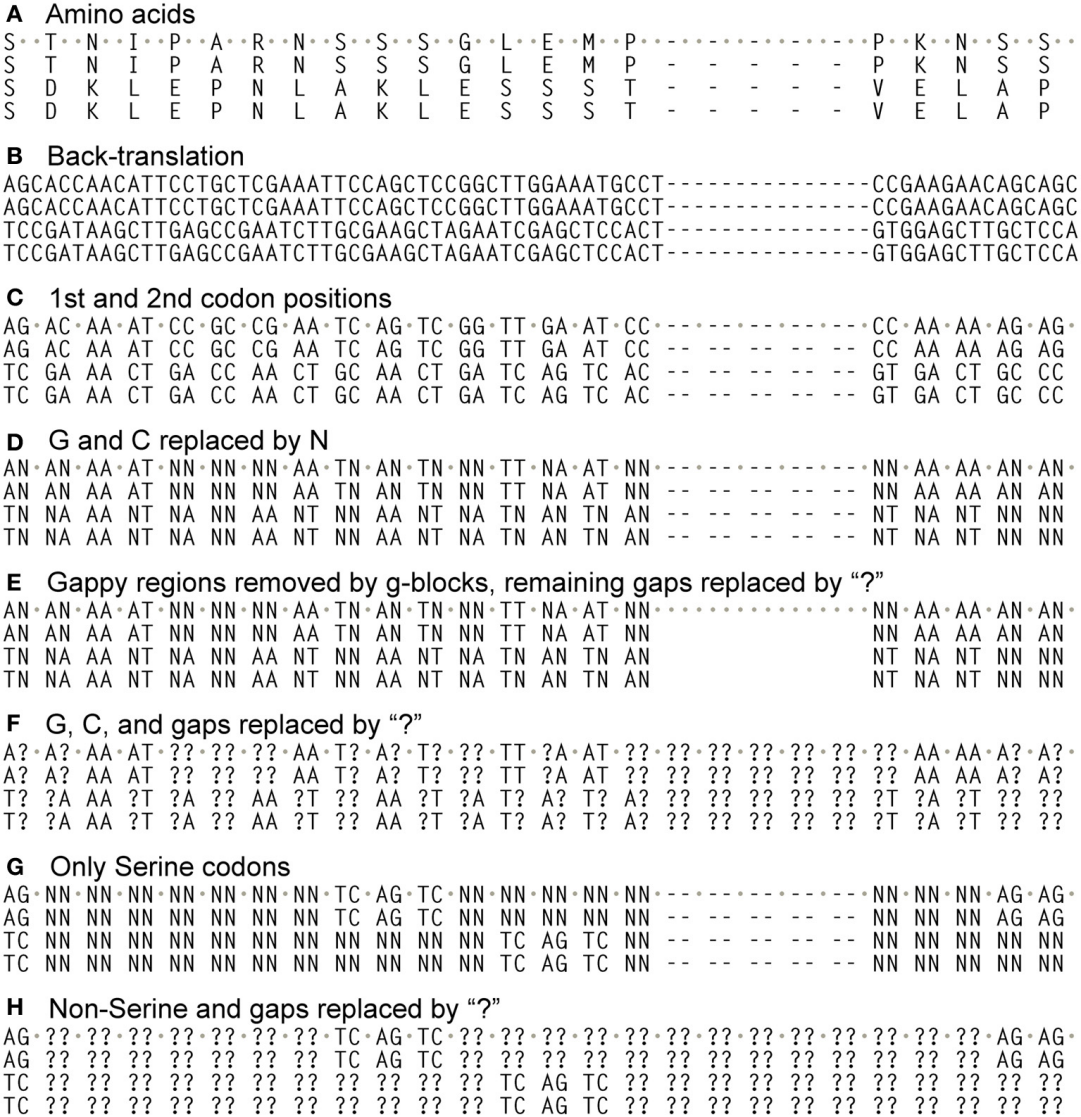
**Different alignments used in this study**. Alignments began by translating coding sequences and aligning them with ClustalW2 (“gapopen” option set to 15), and then back-translating them into nucleotides **(A,B)**. From these only the 1st and 2nd codon positions were taken **(C)**, and then all G and C nucleotides converted to “N” **(D)**, which is read as “any nucleotide” in the phylogenetic program TNT. This alignment then had gappy regions removed by the program Gblocks **(E)** or had all of its GC bases and gaps converted to “?” **(F)**, which is read as “any nucleotide or gap” in TNT. The alignment in B also had all codon positions except those coding for Serine converted to “N”, then the 3rd codon positons removed **(G)**. This also had all Ns and gaps converted to “?” **(H)**.

**Figure 2:**
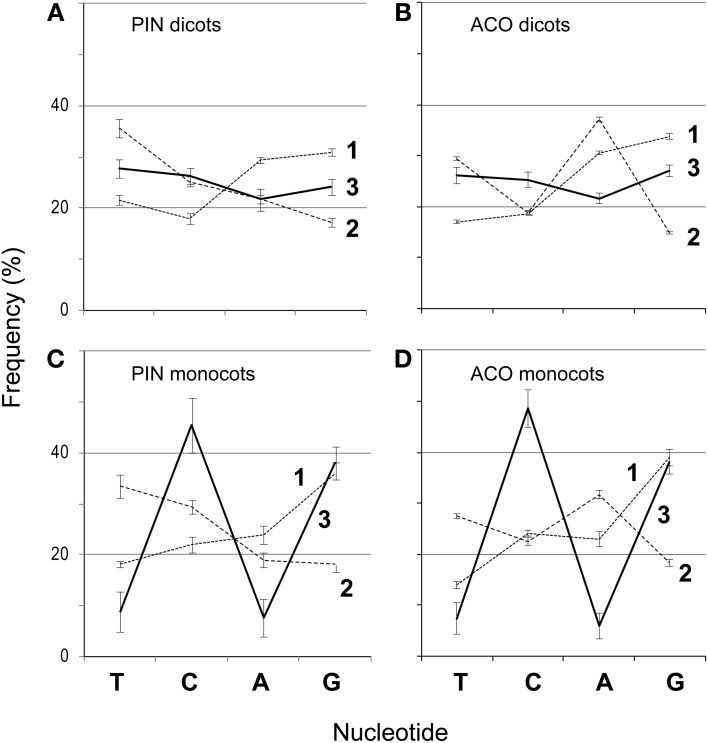
**Frequencies of nucleotides (T, C, A, and G) at each codon position (1–3) for 151 *PIN* and 193 *ACO* genes in dicots (A,B), and monocots (C,D)**. Bars represent 95% confidence intervals.

We aligned the amino acids with ClustalW2 (Larkin et al., 2007) in SeaView (Gouy et al., 2010) (-gapopen parameter set to 15) and then back-translated the amino acids to nucleotides (Figures 1A,B). The resulting nucleotide alignments for the PIN and ACO data sets are available as supplemental material. SeaView retains original nucleotide data through the process of amino acid alignment and thus allows an accurate back-translation after alignment. SeaView also has the alignment program MAFFT (Katoh et al., 2009) implemented, but, with the -gapopen parameter defined, it failed to run with our data set. Clustal misaligned one of the short genes, which was fixed by hand, although tree-searching on the uncorrected version of the alignment resulted in nearly identical topologies. We noticed that monocots tended to have a very high GC content, especially in the third codon position (Figure 2). Thus, using the program Mega v. 4.0.2 (Tamura et al., 2007), we removed the third codon positions of these nucleotide alignments, and in the program BioEdit (Hall, 2007) we replaced G and C with N (Figures 1C,D). Also in BioEdit, we translated nucleotide alignments and replaced all amino acids but Serine with N (Figure 1G); this required several steps, as BioEdit uses a default codon translation for amino acids in back-translation. We also used the program Mesquite (Maddison and Maddison, 2009) to convert fasta files into files that can be read by the phylogenetic program TNT (Goloboff et al., 2008). (Mesquite changes N to “?” automatically when generating TNT files and so these were returned to N using the search-and-replace function in BioEdit.) We used TNT because it clearly reads N as “any nucleotide,” preserving the gap information. We analyzed the same alignments under likelihood bootstrap in the program RAxML (Stamatakis et al., 2008) on the Cipres (Miller et al., 2010) computing cluster. RAxML does not recognize gaps and treats them and N as simply missing data.

For searches in TNT, we used the “new technology” function (which combines several strategies for exploring tree space) with 100 initial builds, and we followed with calculations of bootstrap support using 1000 pseudoreplicates. We took the strict consensus of the shortest trees, which is reported here. For searches of amino acids in POY, we started with 100 Wagner tree builds and conducted SPR and TBR swapping, selecting the shortest trees (with zero-length branches collapsed) and reporting the strict consensus.

Bootstrap support, which is a measure of the redundancy of signal for optimal clades, was not expected to be high, given the nature of these analyses. Single-gene phylogenies with large portions of their content removed to avoid convergence are unlikely to contain enough information to support every clade in resampling analyses, but we do report resampling support for the largest alignments (all 1st and 2nd codon positions, with GC replaced by N).

**Figure 3:**
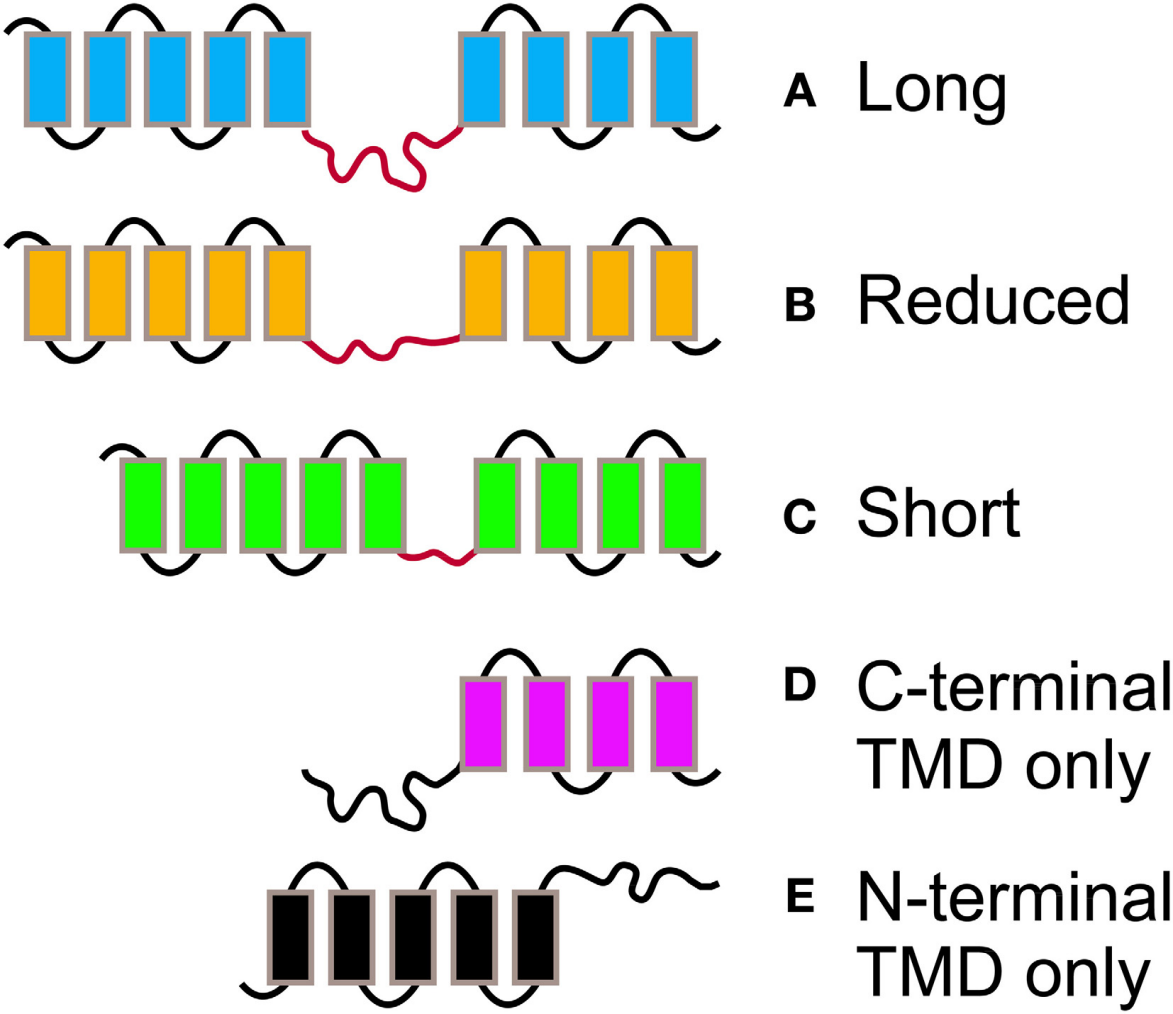
**The different morphologies postulated for PIN proteins**. PIN proteins are classified into 5 groups according to their length and structure. PINs with two complete TMDs and a long **(A)**, reduced **(B)**, or short **(C)** central hydrophylic loop. PINs with reduced protein length and presence of a TMD at the C-terminal **(D)**, or N-Terminal end **(E)** only.

To mimic the way RAxML treats gaps and missing data, we also replaced all Ns and gaps with “?” (Figures 1F,H) and reran them in TNT, which reads “?” as “any nucleotide or gap.” We also removed gappy regions in the program Gblocks (Castresana, 2000) (Figure 1E), replacing the remaining gaps (which were allowed in half of the positions) with N. We also searched the unaligned amino acid sequences in the phylogenetic program POY v. 4 and 5 (Varón et al., 2009), which optimizes the multiple sequence alignment and tree searching simultaneously. When completed, *PIN* trees were uploaded to Mesquite with a character matrix of their protein lengths (Figure 3; coded as 1–5), and parsimony ancestral reconstructions traced over the trees. Characters were treated as unordered.

## Results

**Figure 4:**
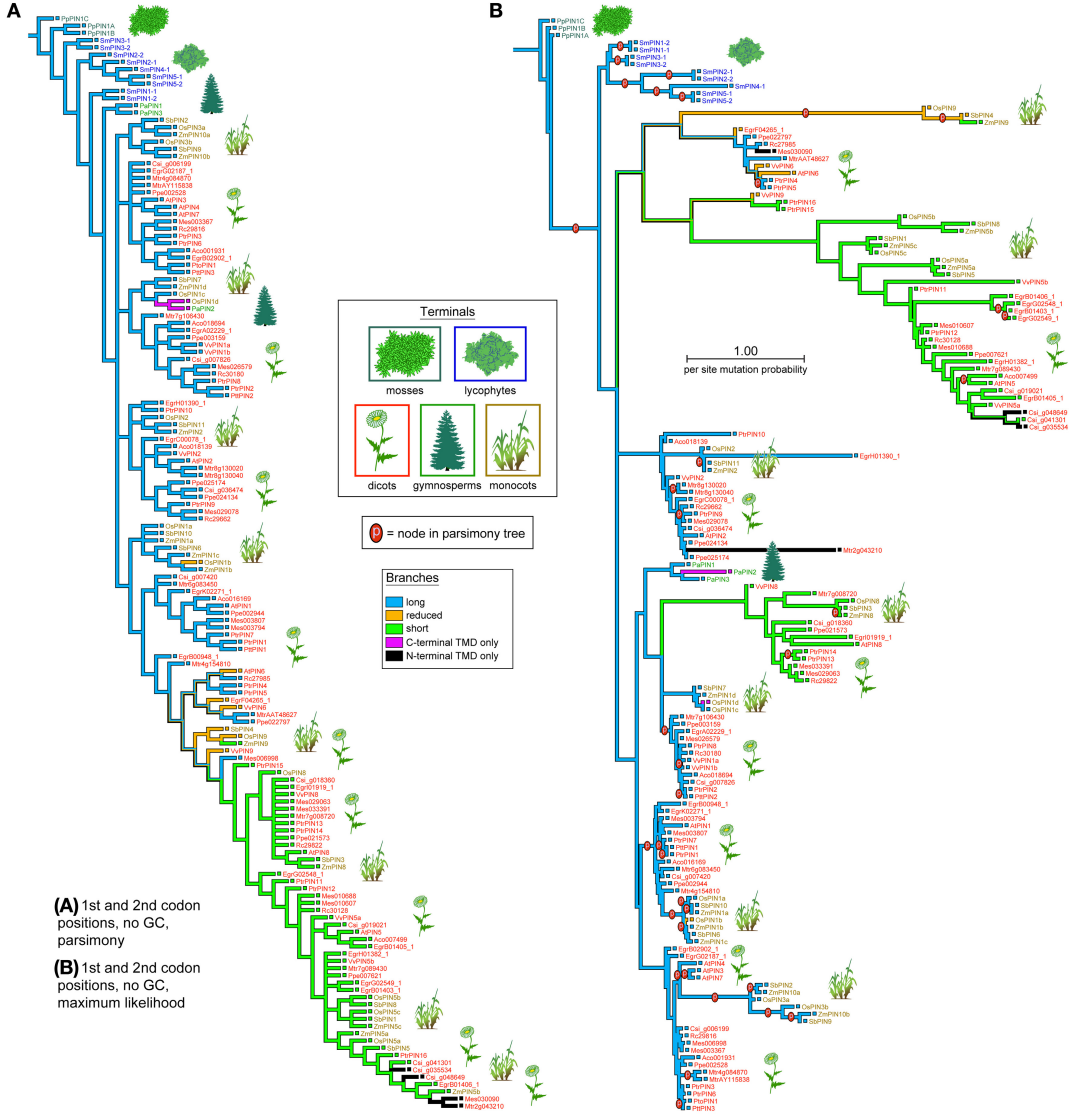
**Phylogeny of the PIN genes, using only the 1st and 2nd codon positions and with al Gs and Cs converted to “N.”** The strict consensus of 85 equally parsimonious trees **(A)**, and the most likely tree under the GTR model **(B)**. Branches are colored according to gene morphology, with parsimony-based historical reconstructions in both trees; equally parsimonious ancestral reconstructions are shown by multi-colored branches. Terminals are colored according to plant taxon, with icons used as guides. Clades recovered under the likelihood optimality criterion **(B)** which were also recovered under parsimony **(A)** are noted with the letter “p.”

**Figure 5:**
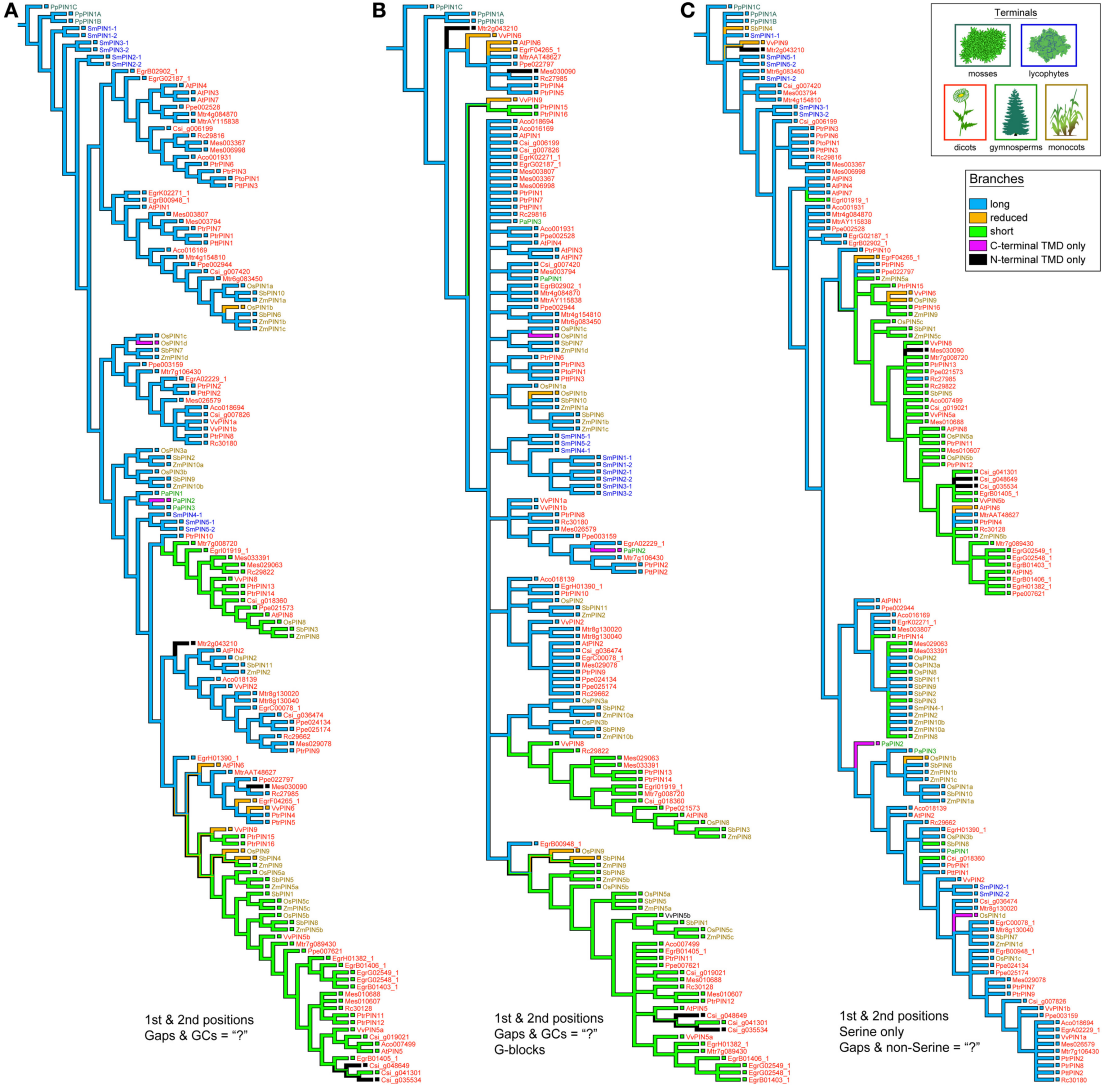
**Strict consensuses of equally parsimonious trees using various alignments of the PIN gene data set with only 1st and 2nd codon positions**. With Gs and Cs removed, the influence of gaps was minimized so as to mimic how gaps and Ns are treated in likelihood programs by replacing them with “?” **(A)**. Alternatively, the alignment had gappy regions removed using the program Gblocks, and then all Gs, Cs, and remaining gaps replaced by “?” **(B)**. A Serine-only alignment was also used, with only 1st and 2nd positions and all gaps and non-Serine postions replaced with “?” **(C)**.

*PIN* phylogenies recovered from a variety of sequence alignments and under parsimony or likelihood reconstructed the evolution of these genes from long to short (Figures 4, 5). The phylogeny recovered with full gap information under parsimony showed a clearer evolution from long, through intermediate, and to short forms, and the short versions were recovered as two clades under likelihood and parsimony when gaps were replaced by “?” or gappy regions removed by Gblocks (Figures 4B, 5A,B). Although several smaller clades remained stable throughout the analysis, and the moss and most of the lycophyte sequences tended to remain as sister to the remaining *PIN* genes, the relationships among the major lineages were generally unresolved. Genes having the trans-membrane domain only on the C-terminal end appeared to have evolved from long-form *PINs*, possibly twice, and those having this domain only on the N-terminal appear to have evolved from short-form genes, perhaps more than once. Monocot *PIN*s were recovered mostly in small stable clades mixed among the dicot genes. Lycophyte genes were monophyletic under likelihood and under parsimony when the alignment was trimmed by Gblocks (Figure 5B); otherwise they tended to form a paraphyletic grade at the base of the tree, near the *Pyscomitrella patens* (moss) genes, or several small clades, only some of which were near the moss genes. Gymnosperm genes were found monophyletic only under likelihood (Figure 4B), and at the base of the tree, after the lycophytes, under parsimony (Figure 4A).

**Figure 6:**
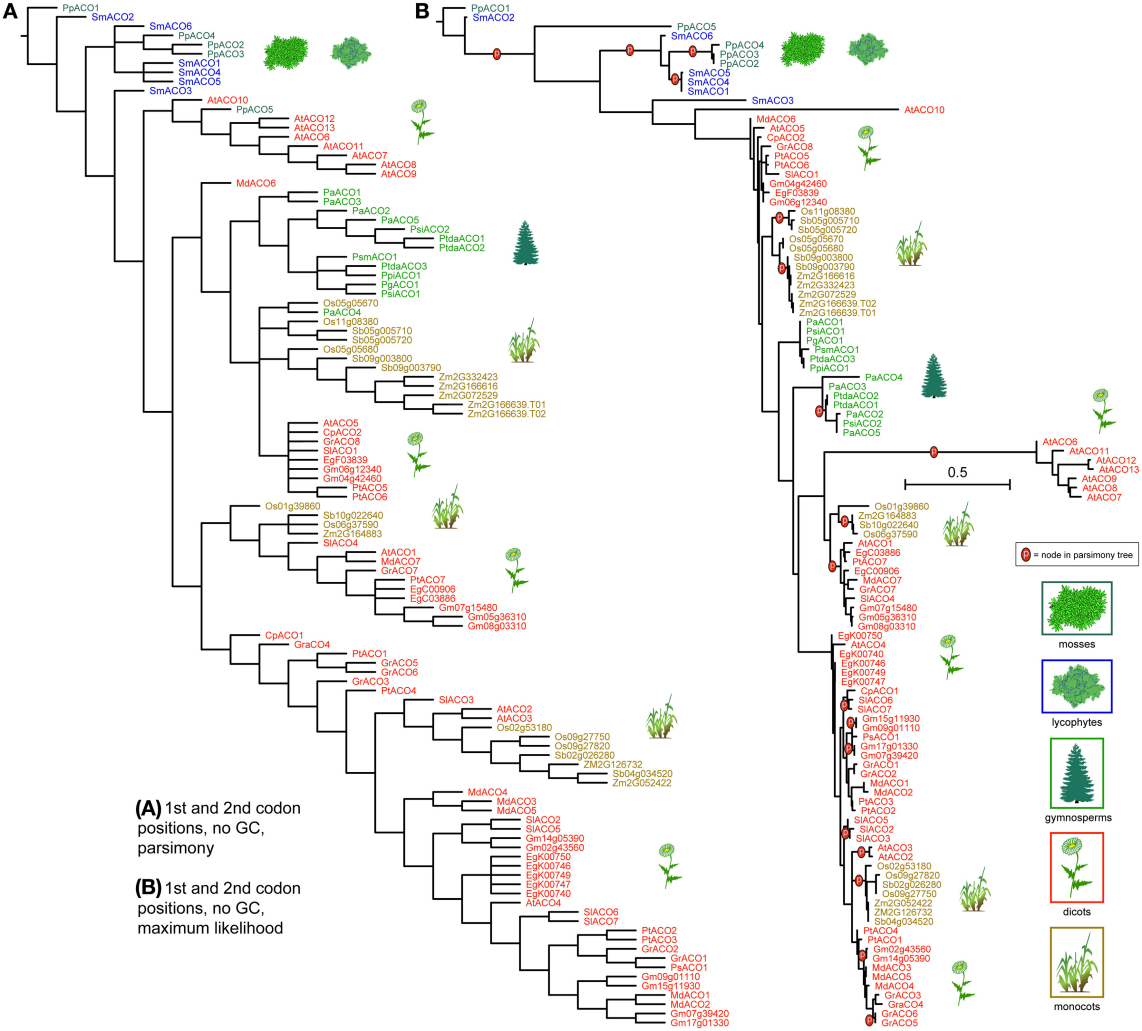
**Phylogeny of the *ACO* genes, using only the 1st and 2nd codon positions and with al Gs and Cs converted to “N.”** The strict consensus of 42 equally parsimonious trees is shown in **(A)**, and the most likely tree under the GTR model is shown in **(B)**. Terminals are colored according to plant taxon. Clades recovered under the likelihood optimality criterion **(B)** which were also recovered under parsimony **(A)** are noted with the letter “p.” Scale bar equals per-site mutation probability.

*ACO* genes clustered more distinctly by taxonomic group, under both parsimony and likelihood, except for the moss and lycophyte genes, which were mixed at the base of the tree (Figure 6). Gymnosperm *ACO*s were recovered as closely related but not near the basal plants. Under parsimony gymnosperms *ACO*s were recovered as monophyletic, with the exception of the Norway spruce gene *PaABO4*, and under likelihood they were recovered in two clades that formed a paraphyletic grade between two angiosperm diversifications. In both analysis, *ACO*s of monocotyledonous species (*Oryza sativa*, *Sorghum bicolor*, and *Zea mays*) cluster into three groups of closely related copies; under likelihood these groups are each monophyletic. Genes resulting from duplications are recovered as closest in both analysis (for example *Sb05g005710* and *Sb05g005720*). *ACO*s from dicotyledonous species form several clades or paraphyletic grades, each containing copies from a mixture of species, except one which contains exclusively *Arabidopsis* sequences. As in monocots, dicot species that underwent relatively recent whole genome duplications (*Malus domestica*, *Populus thricocarpa*) present tightly related gene copies with high sequence similarity.

**Figure 7:**
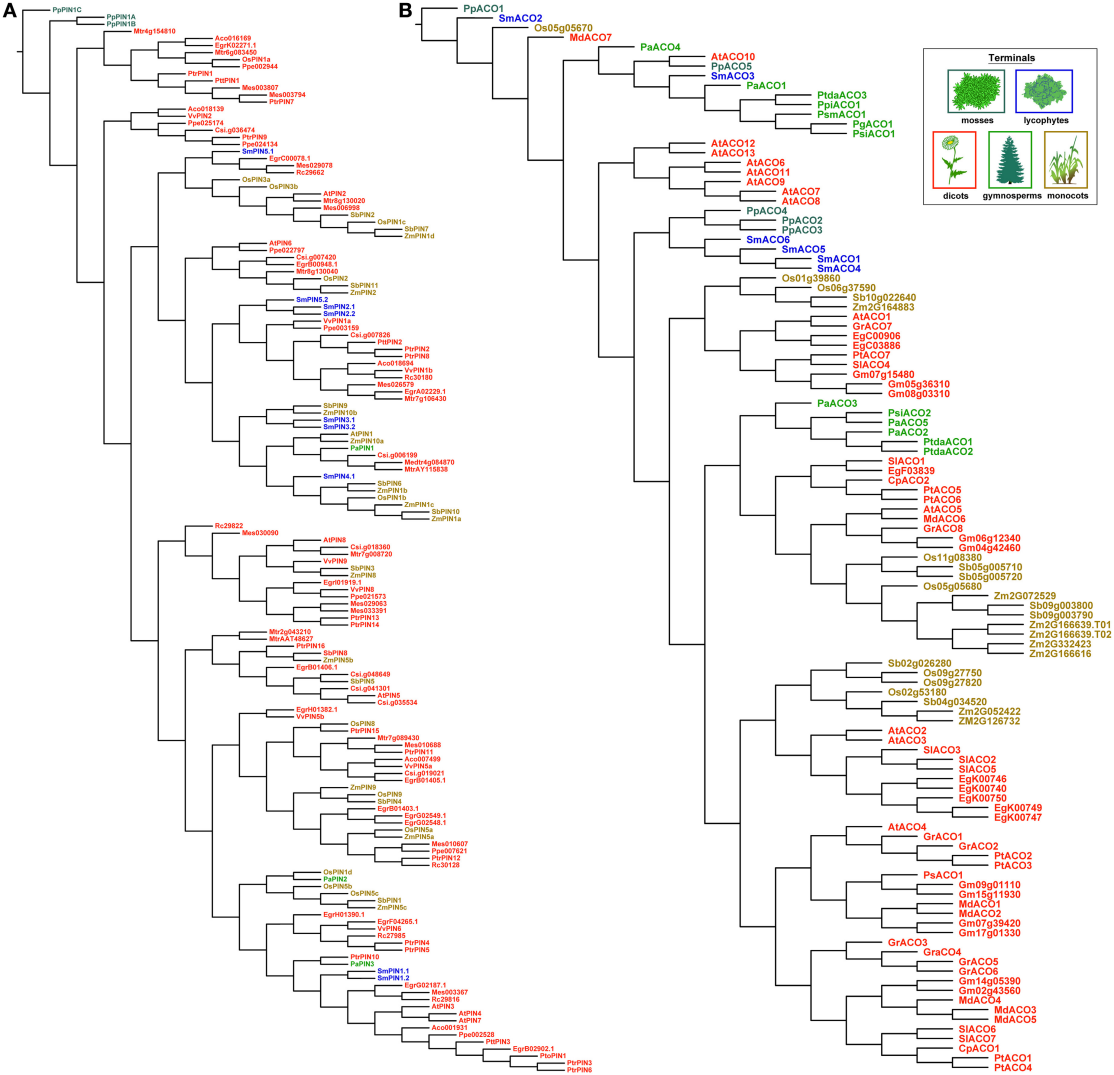
**The most parsimonious reconstructions of the *PIN* (A) and *ACO* (B) gene family histories, using amino acid sequences analyzed using dynamic homology (alignment and tree-search optimized simultaneously)**. Terminals are colored according to plant taxon.

Trees recovered under dynamic homology using amino acids were less organized by taxonomic group than those recovered using the nucleotide alignments above (Figure 7). With *PIN* genes, copies from the moss *P. patens* remained sister to all other *PINs*, but the lycophyte copies were recovered throughout the rest of the tree. This would make any ancestral reconstruction of gene length ambiguous, as the lycophyte copies are classified as long. With the *ACOs*, the mixing of gene copies by taxonomic group occurred mostly at the base of the tree, although it was more complete.

Tree lengths, numbers, and likelihoods are provided in Table 3. The full alignment of 1st and 2nd codon positions, with all GC content replaced by N resulted in a large number of equally parsimonious trees for *PINs* and *ACOs*, mostly due to equally parsimonious resolutions among the main lineages in *PINs*, and among members of small, derived clades in *ACOs*. These unstable regions of each gene family's phylogeny also received little bootstrap support (Figures S1, S2).

**Table 3:** **The number of most parsimonious trees found and their lengths, or the log likelihoods, for each of the alignments searched**.

**Gene family**	Alignment and figure^*^	**No. trees**	**Lengths**	**Likelihood**
*PIN*	D (Figure 4A)	85	11,745	
	D (Figure 4B)			−12279.865284
	F (Figure 5A)	1	2960	
	E (Figure 5B)	2	843	
	H (Figure 5C)	1	494	
	A (Figure 7A)	1	22,706	
*ACO*	D (Figure 6A)	42	4215	
	D (Figure 6B)			−6064.187817
	A (Figure 7B)	1	8688	

Alignment codes (D, F, H, etc.) follow those illustrated in Figure 1, and the figure showing the resulting tree is noted.

* Alignments, illustrated in Figure 1:

(A) Amino acids.

(D) G and C replaced by N.

(E) Gappy regions removed by g-blocks, remaining gaps replaced by “?.”

(F) G, C, and gaps replaced by “?.”

(H) Non-Serine and gaps replaced by “?.”

## Discussion

We find strong support for shorter *PIN*s evolving during the diversification of angiosperms, and the basal plants (here represented by the moss *Physcomitrella patens* and lycophyte *Selaginella moellendorfii*) retaining only long *PIN*s. Whether originating more than once or not, short *PINs* have evolved more recently and their number increased in monocots and dicots following genome duplications in species such as *Oryza Sativa*, *Zea mays*, *Populus trichocarpa*. This finding contradicts that of Viaene et al. (2013). The difference results from how our phylogenies are rooted, and rooting rests homology assessments, alignment methods, the way phylogenetic programs handle missing data, and assumptions about whether primitive plants are more likely to retain primitive gene copies. There are several tests for homology (Patterson, 1988), and the only one presently available for analyzing gene families is similarity; thus for highly dissimilar sequences some justification should be offered for their inclusion. To illustrate how divergent the outgroups of Viaene et al. (2013) are, their supposed *PIN* homolog from ants has a shorter uncorrected *p*-distance relative to the ingroups when reversed than in its original direction. Homology for the animal sequences cannot be justified based on auxin transport, since animals do not have auxin, so it seems likely such sequences are simply not homologous. For such divergent sequences, even if homologous, they will generate large gappy sections in the alignment, only some of which can be removed by hand, and they will likely root randomly. Another source of alignment difficulties is the partial nature of the algal *PIN* sequences which Viaene et al. (2013) took from ESTs. If the phylogenetic programs they employed treated gaps and missing data the same, algal sequences among the outgroups would be inclined to pull short *PIN* genes to the base of the tree. Indeed, it is not surprising that non-homologous and partial sequences in the outgroups attracted the strange and truncated PpPIN1D sequence to the outside of the ingroups—more through shared exclusion than similarity—thus strengthening the appearance of a short-to-long evolution of *PIN* genes.

In the absence of a suitable outgroup for plant PIN genes, we have simply made the assumption that plants recovered as sister to all the others (what one might call “primitive” or “basal” plants) carry gene copies that are also likely to be sister to all the others. This may not be correct, as evidenced by the placement of some gymnosperm copies as more derived than angiosperm copies. However, perhaps the most stable and supported relationship we recovered with *PINs* and *ACOs* was the clear distinction between moss (and usually lycophyte) copies and all the others. Thus, these gene families either diversified very early, and mosses and lycophytes retained only the most derived copies, or, more parsimoniously, the gene families simply diversified after the rise of spermatophytes. In any case, we note that the reconstruction of a long-to-short evolution of *PIN* genes is not merely the result of putting moss copies as the outgroup, for even in generally unresolved trees, like the one recovered from the alignment trimmed of gaps by Gblocks (Figure 5B), the main clades of short-form *PINs* are closely related to each other and are derived from angiosperm long forms. Only rerooting specifically by a short-form copy would change this, and in reconstructions with two origins of short-form copies, one clade would remain derived.

A recent phylogenetic analysis of *PIN* genes done independently of the analysis here (Bennett et al., 2014) uses a similar methodological approach and obtains results that are broadly congruent with our previous analysis (Carraro et al., 2012) and the analysis here. Bennett et al. (2014) use nucleotide sequences and root by bryophytes, and they recover the odd *PpPIND* in a derived position on a long branch. They also find multiple, later origins for short (or more specifically, what they term “non-canonical”) forms of *PIN* genes. It thus appears that whether improvements are made to *PIN* phylogenies by adding more sequences (Bennett et al., 2014) or excluding sources of convergence in the data, as we do here, *PIN* genes increasingly seem to have undergone shortening events multiple times.

In our phylogenies it appears that most modern *ACOs* arose subsequent to the monocot-dicot split [140–150 Ma ago, during the late Jurassic-early Cretaceous (Chaw et al., 2004)], but *PIN* genes diversified much earlier, as evidenced by their more thorough historical mixture of monocot and dicot copies. A broad diversification of *ACOs* during the Mesozoic is later than was previously hypothesized by John (1997), who did not consider them present in primitive land plants and believed their appearance was necessitated by droughts at the end of certain Permian periods (the Devonian at 360 Ma ago and the Carboniferous at 300 Ma ago). It appears that multiple ACO copies were present during the Permian and before the split between gymnosperms and angiosperms, and even before the split between mosses and lycophytes, but the later proliferation of copies in angiosperms requires a new ecological driver besides droughts in the Permian.

Gymnosperm *PINs* and *ACOs* appear to have derived from angiosperm gene copies. This is not corrected simply by using a gymnosperm root, since that renders moss and lycophyte copies derived. Rather it indicates that for both *PIN*s and *ACO*s the ancestral copy in gymnosperms was a more derived copy than some of the copies inherited and retained in the ancestral angiosperm. A very limited number of gymnosperm sequences are available for both gene families, so the possibility remains that the history of gymnosperm sequences will become clearer as more of them are included in future analyses.

Amino acid sequences should present problems for historical reconstruction (Simmons, 2000), despite their popularity in plant gene family phylogenies. Genetic code degeneracy and selection pressure on protein function are sources of convergence, and although amino acids may correct for back-mutations in the third codon position, which can be another source of convergence, ignoring the third codon position removes information on recent divergences. Using amino acids with phenetic tree-building methods like Neighbor-Joining (algorithms that cluster sequences by overall similarity) (Saitou and Nei, 1987) will likely amplify convergence in amino acids, and using them with probabilistic optimality criteria requires a model of evolution both for the alignment step and tree-searching. We find here that trees made with amino acids and the most agnostic cladistic method for optimizing alignment and tree-searching (dynamic homology) produced trees with little in their favor relative to the trees made from GC-free nucleotide alignments, and we would not recommend using amino acids for future historical studies of gene families.

Although a few monocot *PIN* and *ACO* seqeunces do not have a high GC content, it seems likely that this quality is the result of an ongoing substitution bias and thus a source of misleading, convergent signal. Monocot copies are not monophyletic, and almost all of them present very high GC content, which argues against this being the result of a historical event no longer maintained in monocots. For example, in *PINs* almost half of the monocot copies have GC content at the third codon position above 50%, and all but four are over 40%. We notice in Meister and Barow's survey (2007) that monocots in general have statistically significantly higher GC content than dicots (using a *t*-test of the GC percentages, *p* < 0.001), and that they attain a maximum content around 50%, about 10% higher than the maximum GC content of dicots. However on average monocot genomes have only about 1% more GC content than dicots. We notice that the most GC-rich species in Meister and Barow (2007) are the grasses, which constitute all the monocots in our data set. Given how strong this bias appears in grass *ACOs* and *PINs*, it was perhaps trivial for us to retrieve monocot clades in our previous PIN phylogeny (Carraro et al., 2012), which was based on all three codon positions and did not have GC content removed. However, we still recover most monocots together (either still in clades or paraphyletic grades) and in the same combinations here as before. For example, the monocot group of nine short-form genes that includes OsPIN5b and SbPIN5 was recovered both previously and here (Figure 4B), but previously these were monopheletic, and here they form a grade out of which diversifies a clade of short-form and N-terminal-TMD-only genes. Even using the alignment that had only the first and second positions of serine, which should be immune from a GC mutational bias, we recovered clusters of monocots, a close relationship among primitive land plants, and a clade of mostly short PIN genes (Figure 5C).

Here we present new phylogenies for *PIN* and *ACO* genes, after working to improve the methods used to reconstruct the histories of gene families. First, we avoid the use of amino acids and distance (phenetic) algorithms, which have the potential to convey and amplify homoplasy. Next, in a further attempt to avoid homoplasy among genes with similar lengths and GC content, we avoid the use of indels and treat Gs and Cs or their transformations as missing data. We root trees by gene copies found in bryophytes, and we exclude sequences which are not clearly homologous. The results suggest an evolution from long to short *PIN*s, perhaps multiple times, and a diversification of *ACO*s mostly after the dicot-monocot split. More sequences from a wider taxonomic range for these gene families are welcome for the continued development of their phylogenetic hypotheses and a deeper understanding of their histories.

### Conflict of interest statement

The authors declare that the research was conducted in the absence of any commercial or financial relationships that could be construed as a potential conflict of interest.
