# Supplementary material for: A novel phylogeny and morphological reconstruction of the PIN genes and first phylogeny of the ACC-oxidases (ACOs)
Source: Front Plant Sci. 2014 Jun 24;5:296. doi: 10.3389/fpls.2014.00296 (PMC4071234; doi:10.3389/fpls.2014.00296)
Supplement: Supplementary file 1 [file Presentation1.ZIP › Supplementary_Figure_1.pdf]

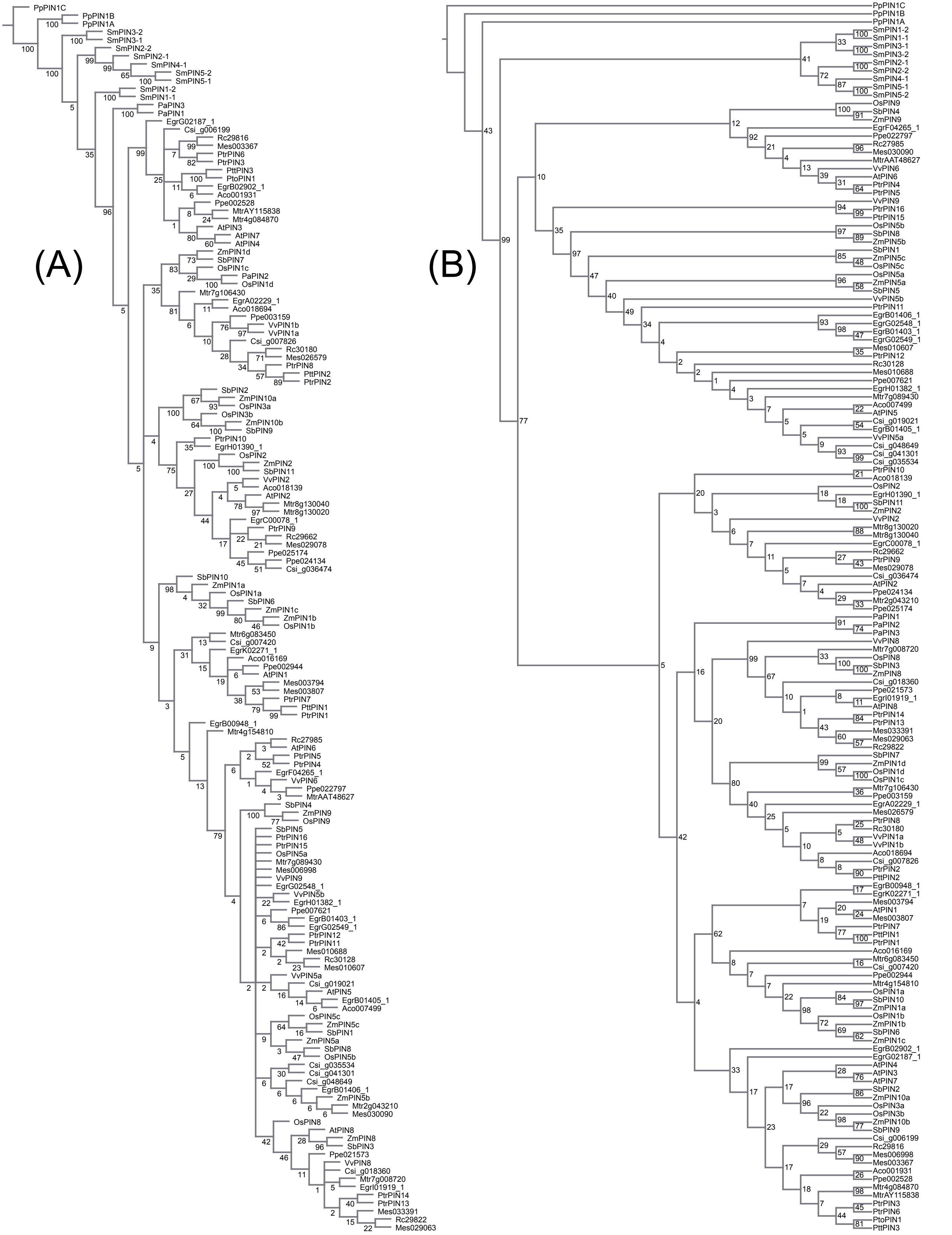

Figure S1. Bootstrap resampling support values for the strict consensus of the most parsimonious trees (A) and the most likely tree (B) of the *PIN* gene family. Trees are the same as those shown in Fig. 4. Parsimony bootstrap values were calculated in TNT using 1,000 pseudoreplicates, and likelihood bootstraps were calculated in RAxML.
